# Protein kinase CK2-dependent aerobic glycolysis-induced lactate dehydrogenase A enhances the migration and invasion of cancer cells

**DOI:** 10.1038/s41598-019-41852-4

**Published:** 2019-03-29

**Authors:** Dae-Kyun Im, Heesun Cheong, Jong Suk Lee, Min-Kyu Oh, Kyung Mi Yang

**Affiliations:** 1 Department of Chemical and Biological Engineering, Korea University, Seoul, 02841 Korea; 2 Division of Cancer Biology, Research Institute, National Cancer Center, Goyang, 10408 Republic of Korea; 3 Biocenter, Gyeonggido Business and Science Accelerator (GBSA), Suwon, Gyeonggi-do 16229 Republic of Korea; 4 Department of Biochemistry and Molecular Biology, Yonsei University College of Medicine, 50-1 Yonsei-ro, Seodaemun-gu, Seoul 03722 Republic of Korea

## Abstract

We investigated the intracellular metabolic fluxes of protein kinase CK2-activating (Cα OE) cells and role of lactate dehydrogenase A (LDHA) as a contributor of tumorigenesis after reprogrammed glucose metabolism. Facilitated aerobic glycolysis was confirmed via isotope tracer analysis, in which ^13^C_6_-Glc or ^13^C_5_-Gln was added to the media, following which metabolites converted from Cα OE cells were identified. We found a greater decrease in cell survival, colony-forming ability, migration, and Cα OE cell invasion under glucose (Glc)-depletion conditions than under glutamine (Gln)-depletion conditions. Cancer cell migration and invasion increased due to LDHA elevation of the altered metabolic axis driven by activated CK2. FX11 treatment and LDHA knockdown suppressed migration and invasion through ROS generation, but this was partially reversed by the antioxidant *N*-acetylcysteine (NAC). Moreover, LDHA inhibition decreased tumor growth in a mouse xenograft model transplanted with Cα OE cells. Finally, we concluded that LDHA is an excellent metabolic target for tumor therapy, based on CK2α derived aerobic glycolysis.

## Introduction

Reprogramming of energy metabolism can be a functional driver of growth and progression in tumors^1^. Adaptation to metabolic alterations in tumors is beyond the process of aerobic glycolysis, including the balancing of macro-molecular synthesis and redox homeostasis^2,3^. An increasing number of studies have examined relevant specific oncogenes by identifying novel biosynthetic metabolic pathways in cancer cells^4^. Notably, a new strategy has been reported to target glucose metabolism mediated acidosis for colorectal cancer therapy^5^.

Glucose (Glc) and glutamine (Gln) support the biological hallmarks of malignancy^6,7^. They are the main fuel for cancer cell growth and are used for biosynthesis and energy generation. Particularly, cancer cells fervently absorb Glc during aerobic metabolism, which is then metabolized to pyruvate, followed by conversion preferably to lactate by lactate dehydrogenase A (LDHA), as opposed to entering the mitochondrial tricarboxylic acid (TCA) cycle^8^. LDHA is predominant in skeletal muscles, while lactate dehydrogenase B (LDHB) is expressed mainly in the heart muscle^9^. Furthermore, because the expression of LDHA in human cancer tissues is higher than that in normal tissues, LDHA is considered a promising target for cancer diagnosis and therapy^10^. It was reported that high LDHA expression in human lung carcinoma and hepatocellular carcinoma markedly increased the invasive potential and was associated with the generation of lactate^11,12^. Inhibiting LDHA weakens the Warburg effect and increases the mitochondrial membrane potential, causing cytoskeletal remodeling, which in turn increases the generation of reactive oxygen species (ROS) and oxidative stress, leading to cell death^13,14^.

Glycolysis and mitochondrial respiration are tightly coupled processes^15^. They have been reported to facilitate the cancer stem cell phenotype, angiogenesis, migration, and immune evasion by influencing the cancer microenvironment via accumulated lactate^9,16^. If the conversion of pyruvate to lactate is reduced, cells excessively use the oxidative phosphorylation flux as a metabolic pathway to generate ATP, resulting in ROS due to oxidative stress, eventually leading to cell death^14^. A low-to-medium level of ROS has been shown to facilitate angiogenesis by increasing cell proliferation and survival and inducing the expression of stress-responsive genes^17,18^. In contrast, a high level of ROS facilitates aging and DNA damage, eventually leading to apoptosis^19^. FX11 (an NAD^+^ synthesis inhibitor) could induce oxidative stress, causing lymphatic regression. However, it has been shown that the antioxidant *N*-acetylcysteine (NAC) rescued cell proliferation that was inhibited by ROS, which was generated as a byproduct in the mitochondria^14^.

Protein kinase CK2 is a serine/threonine kinase involved in various biological processes related to cell proliferation and survival^20,21^. We previously reported that CK2 activity was high in anoikis-resistant esophageal cancer cells^22^. Elevated levels of CK2 expression or activity have been reported in many cancer types and are associated with poor prognosis^23^. CK2α also induced epithelial mesenchymal transition (EMT) in colon cancer cell lines such as HT29 and SW620, and the overexpressed cells became more proliferative than the controls. CK2α knockdown in bladder cancer cells reduced tumor aerobic glycolysis and suppressed tumorigenicity by reducing the phosphorylation of AKT^24^. A recent study suggested that because oncogenes regulate important metabolic flux and metabolism signaling pathways, those oncogenes should be promising targets for precision cancer therapy only if the regulation of cancer metabolism by the oncogenes is demonstrated^25,26^. However, the mechanisms of cancer cell proliferation and metastasis via the reprogramming of the glucose metabolism pathway by increased CK2 activity are still unclear.

In the present study, we have investigated the intracellular metabolic fluxes and role of LDHA as a critical factor of migration and invasion in CK2α-activated cells.

## Methods

### Cell culture

HT29, HT29-Cα OE, SW620, SW620-Cα OE, TE2, HCE4 and stable transfectant cells were cultured in Dulbecco’s modified Eagle medium (DMEM; Gibco Laboratories, Gaithersburg, MD, USA) containing 10% fetal bovine serum (FBS; HyClone Laboratories, Inc., Logan, UT, USA), 100 µg/ml streptomycin (Gibco), and 100 units/ml penicillin (Gibco) in a 5% CO_2_ incubator at 37 °C. Gastric cancer cells were cultured in RPMI 1640 medium with 10% FBS. The medium used for the Glc-labeled experiment was Glc-free, pyruvate-free DMEM containing 10% FBS and 1 g/L U-^13^C_6_-Glc (99% purity; Cambridge Isotope Laboratories, Inc., Cambridge, MA, USA). The medium used for the Gln-labeled experiment was Gln-free DMEM containing 10% FBS and 4 mM U-^13^C_5_-Gln (99% purity; Cambridge Isotope Laboratories, Inc.).

### Generation of stable cell lines

To obtain the lentiviral supernatant, the control vector and Lentipgk - mCMV - HA - CK2α IRESpuro plasmid were purchased from Macrogen Inc (Seoul, Korea) and cotransfected into 293 T cells with the packaging plasmid. To obtain stable single-colony transformants, puromycin (2 µg/mL) resistant clones were picked and separated on dishes containing the selection medium with puromycin. The individual clones used for various assays were maintained in cultures in the presence of the antibiotic puromycin (1 µg/mL). Protein expression in each clone was determined by western blots.

### Sample preparation

Intracellular metabolites were extracted by two-phase extraction, as described previously^27^. The samples were vacuum-dried at 24 °C and stored at −70 °C until gas chromatography (GC)-electrospray ionization (EI)-mass spectrometry (MS) analysis. The metabolite samples were subjected to two-stage chemical derivatization for GC-EI-MS analysis. 50 μl of 2 wt% hydroxylamine hydrochloride in pyridine was added to the sample, and the mixture was heated at 70 °C for 50 min. After cooling to 24 °C, 80 μl of *N*-methyl-*N*-(trimethylsilyl) trifluoroacetamide with 1% trimethylchlorosilane (Sigma-Aldrich, St. Louis, MO, USA) was added to the mixture and heated at 70 °C for 50 min for pyruvate and palmitate analysis; 80 μl of *N*-tert-butyldimethylsilyl-*N*-methyltrifluoroacetamide with 1% tert-butyldimethyl chlorosilane (Sigma-Aldrich) was added to the mixture and heated at 70 °C for 50 min for lactate, citrate, malate, fumarate, serine, and glycine analysis.

### GC-EI-MS analysis

The derivatized intracellular metabolite samples were analyzed using a Bruker 450-GC instrument coupled with the Bruker 300-MS single quadruple mass spectrometer (Bruker, Inc. Billerica, MA, USA). The system used a silica capillary column DB-5ms (30 m × 0.25 mm ID, 0.25-μm film thickness, J&W Scientific, Folsom, CA, USA). Helium (99.999% purity) was used as a carrier gas, at a flow rate of 1.0 ml min^−1^. The column oven temperature was initially kept at 70 °C for 1 min, increased to 80 °C at a rate of 2 °C min^−1^ and maintained for 1 min, increased to 200 °C at a rate of 6 °C min^−1^ and maintained for 3 min, and then increased to 270 °C at a rate of 15 °C min^−1^ and maintained for 5 min. The temperatures for the front inlet, transfer line, and ion source were 270, 250, and 200 °C, respectively. The injected volume for each sample was 1 μl in a 1:15 split ratio mode. The mass spectrometry data were acquired in the full-scan mode over an m/z range of 50–800 for metabolite identification and single-ion monitoring mode for isotopomer analysis after a solvent delay of 7 min.

### Colony-forming assay

Colony formation was analyzed using 2 × 10^4^ cells in 6-well plates. The cells were starved of Glc or Gln for 1 week. Colonies were stained with 1% sulforhodamine B (SRB) for 5 min, dried, and photographed. The bound dye was eluted with 10 mM Tris-HCl (pH 7) and detected at 510 nm.

### Chemotactic transwell assay

Cell migration and invasion were measured in 24-well chamber plates with 8-µm pore size polycarbonate membrane filter inserts (Millipore Chemicon, Billerica, MA, USA). The chambers were rehydrated for 1 h in serum-free medium. Cell invasion was determined using Basement Membrane Matrix (BD Biosciences, Franklin Lakes, NJ, USA) pre-coated with matrigel. Complete medium containing 10% FBS served as a chemoattractant in the bottom chamber and 2 × 10^5^ cells/ml were incubated for 48 or 72 h. Migrated or invaded cells at the bottom surface of the membrane were stained with 1% SRB or methylene blue for 10 min, dried, and photographed. The bound dye was eluted with 10 mM Tris-HCl (pH 7.0). The ability of cell migration or invasion was determined by measuring the absorbance at 510 nm using a microplate reader (SpectraMax 190^®^; Molecular Devices Corp., Sunnyvale, CA, USA).

### Measurement of ROS levels

Intracellular ROS levels were measured using DCFDA (2′,7′-dichlorodihydrofluorescein diacetate) Cellular ROS Detection Assay Kit (ABcam, Cambridge, UK), according to the manufacturer’s instructions. Mitochondria-specific superoxide radicals were assessed by MitoSOX Red indicator (Invitrogen Co., Carlsbad, CA, USA). Cells were loaded with MitoSOX at a 5 μM concentration and incubated for 10 min at 37 °C. Cellular red fluorescence intensity was detected using an Olympus IX71 fluorescence microscope. The ImageJ software was used to quantify the intensity of ROS fluorescence.

### LDHA knockout using CRISPR/Cas9 system

The sgRNA target sequence (GGTGTAAGTATAGCCTCCTG) was designed using Genscript software (genscript.com/gRNA-design-tool). sgRNA was cloned into the lentiCRISPR v2 vector (Addgene, Cambridge, MA, USA). Generation of CRISPR/Cas9-mediated LDHA knockout (KO) lentivirus was conducted using a lentiviral packaging system (pMD2.g and psPAX2). Transduced Cα OE cells with lentivirus were selected using blasticidin.

### PI/Annexin V analysis

Apoptotic cells were analyzed using Annexin V-FITC and propidium iodide (PI) dye (BD Biosciences, San Jose, CA), according to the manufacturer’s instructions. The data were analyzed using the FlowJo software (version 7.6.1; FlowJo LLC, Ashland, OR, USA).

### Western blot analysis

Western blot analysis was performed as previously described^28^. Membranes were immunostained with antibodies specific for the following antigens: LDHA (Cell Signaling Technology, Inc. MA, USA), and β-actin (Sigma Aldrich, St. Louis, MO, USA). The signals were visualized using the chemiluminescence (ECL) detection kit (Amersham Pharmacia Biotech Inc., Uppsala, Sweden).

### Cell cycle analysis

The cell cycle and sub-G1 distributions were determined by staining DNA with PI (10 µg/ml). Cells (3 × 10^5^) were incubated for 24 and 48 h. They were washed in PBS and fixed in 70% ice-cold ethanol for 1 h. The cells were washed again with PBS and then incubated with PI (10 µg/ml), followed by simultaneous treatment with RNase (200 µg/ml) at 37 °C for 1 h. The percentages of cells in each phase of the cell cycle or having sub-G1 DNA contents were measured with a LSRII flow cytometer (Becton Dickinson, San Jose, CA, USA) by plotting at least 20,000 events per sample. Data were analyzed by using FlowJo software (version 7.6.1; FlowJo LLC, OR).

### Determination of kinase activity

Intracellular CK2 kinase activity was determined as previously described^22^.

### Xenograft experiment

LDHA inhibitor FX11, which was purchased from Millipore Chemicon, was initially dissolved in 2% DMSO. All experimental procedures performed in this study followed the ethical guidelines for animal studies and were approved by the Institutional Animal Care and Use Committee of Yonsei University College of Medicine (IACUC No. 2013-0147-1). Cells (4 × 10^7^) were subcutaneously injected into the right flank of 6-week-old female BALB/c nude mice. When the size of the tumor reached 200 mm^3^ after cell administration, the mice were randomly divided into the control group (SW620, SW620-Cα OE; 2% DMSO, administered intraperitoneally, daily; n = 6) and 2.5 mg/kg FX11-treated SW620-Cα OE group (administered intraperitoneally, daily; n = 7). Intraperitoneal injection (IP) of FX11 or vehicle was performed daily for two weeks. Tumor size was calculated by measuring the length and the width of the tumor using a caliper [tumor volume (mm^3^) = length × width^2^/2]. One week after the final treatment, all mice were sacrificed, and the tumors were removed and photographed.

### Statistical analysis

Data are presented as the mean ± SD for each group. Differences between control groups and treated were analyzed using the Student *t* test or one-way ANOVA. *P* < 0.05 was considered statistically significant. Data shown are representative of duplicate or three independent experiments.

## Results

### Cα OE cells are sensitive to Glc as a carbon source for survival

**Figure 1:**
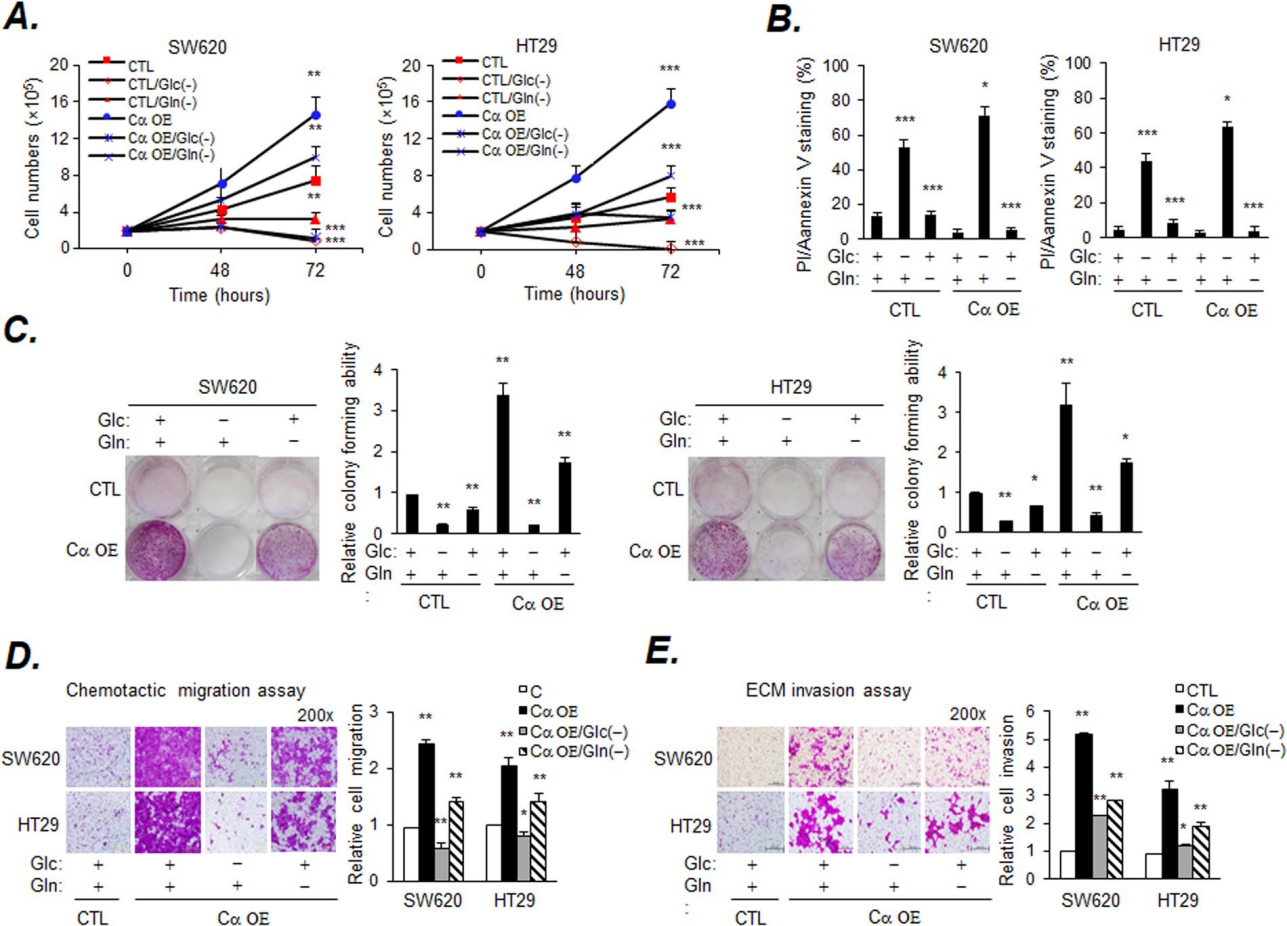
Glucose is necessary for Cα OE cells survival, migration, and invasion. (**A**) The survival of HT29 or SW620 cells is shown; CK2α represents HT29 or SW620 cells transduced with lentivirus expressing CK2α under Glc- or Gln-deprived conditions. Cells (2 × 10^5^) were incubated in Glc- or Gln-free DMEM, and the number of surviving cells were estimated at the indicated time-points. The number of cells was counted using an ADAM automatic Cell Counter. Values are expressed as the mean ± SD (n = 3; **p < 0.01; ***p < 0.001 at 72 h). (**B**) The percentage of cell death was assessed by LSRII FACS analysis. Cells (5 × 10^5^) were cultured under Glc- or Gln-depletion conditions in regular medium for 72 h. (**C**) The colony-forming ability was assessed by SRB staining. Cells (1 × 10^3^) were cultured under Glc- or Gln-depletion conditions in regular medium for one week. (**D** and **E**) Reduced migration and invasion of Cα OE cells in the absence of Glc. Cells (2 × 10^5^/well) were seeded and cultured for 48 h. Migrated and invaded cells were visualized by SRB staining and eluted by 10 mM Tris-HCl buffer. Original magnification, 200×. Values are expressed as the mean ± SD (n = 3; *p < 0.05; **p < 0.01). Original magnification, 200×. Scale bar, 500 µm.

We have previously reported induction of EMT and facilitation of Warburg effect in HT-29 and SW620 colon cancer cells by lentivirus overexpressing the CK2α catalytic subunit^29^. To assess the nutritional requirements of Cα OE cells with regards to a carbon source, cell growth was observed under Glc- and Gln-depletion conditions. The numbers of SW620-Cα OE and HT29-Cα OE cells were markedly lower under Glc-depletion conditions than under Gln depletion conditions. In contrast, the number of Cα OE cells grown under normal conditions was much higher than that of the control (CTL) cells (Fig. 1A). Cell death was assessed under the same conditions using PI/Annexin V staining and was found to be higher in Cα OE cells than in control cells under Glc-depletion conditions (Fig. 1B and Supplemental Fig. S1). Consistent with these findings, the colony-forming ability of Cα OE cells significantly decreased under Glc-depletion conditions (Fig. 1C). The colony of the Gln depleted cells was reduced to some extent. However, the Cα OE cells with the EMT induction phenomenon and fast cell growth showed a substantial reduction in Glc depleted cells, thus it is contended that they are Glc-dependent cancer cell lines. There are more viable cells in the Gln depleted condition than the viable cells in the Glc depleted condition. Cα OE cells have a fast Glc consumption, while Gln consumes minimal for the energy supply of cells. Next, we investigated the degree to which Gln and Glc were involved in malignant phenotypes, such as the migration and invasion of Cα OE cells. Migration, assessed as the ability of cells to move in a direction opposite to the permeable support, was assessed by the transwell assay. Migration and invasion considerably increased in Cα OE cells cultured in complete media, but noticeably decreased under Glc-depletion conditions (Fig. 1D,E). Under Gln-depletion conditions, the effect was moderate.

### CK2α facilitates the Warburg effect and shifts toward pyruvate-to-oxaloacetate and pyruvate-to-acetyl CoA flux in Cα OE cells

**Figure 2:**
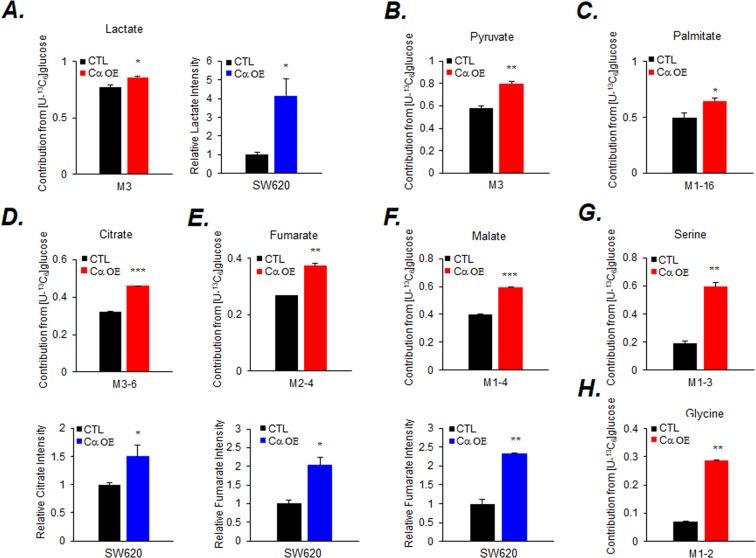
Stable isotope tracer analysis of [U-^13^C]-Glc in SW620-Cα OE cells. Cells (5 × 10^6^) were cultured in Glc-depleted media containing 1 g/L U-^13^C_6_-Glc overnight before extraction. The contribution (red color) and relative intensity (blue color) of the metabolites, (**A**) lactate (M3), (**B**) pyruvate (M3), (**C**) palmitate (M1-16), (**D**) citrate (M3-6), (**E**) fumarate (M2-4), (**F**) malate (M1-4), (**G**) serine (M1-3), and (**H**) glycine (M1-2) in the cell extracts were measured by GC-EI-MS. Values are expressed as the mean ± SD (n = 5; *p < 0.05; **p < 0.01; ***p < 0.001).

**Figure 3:**
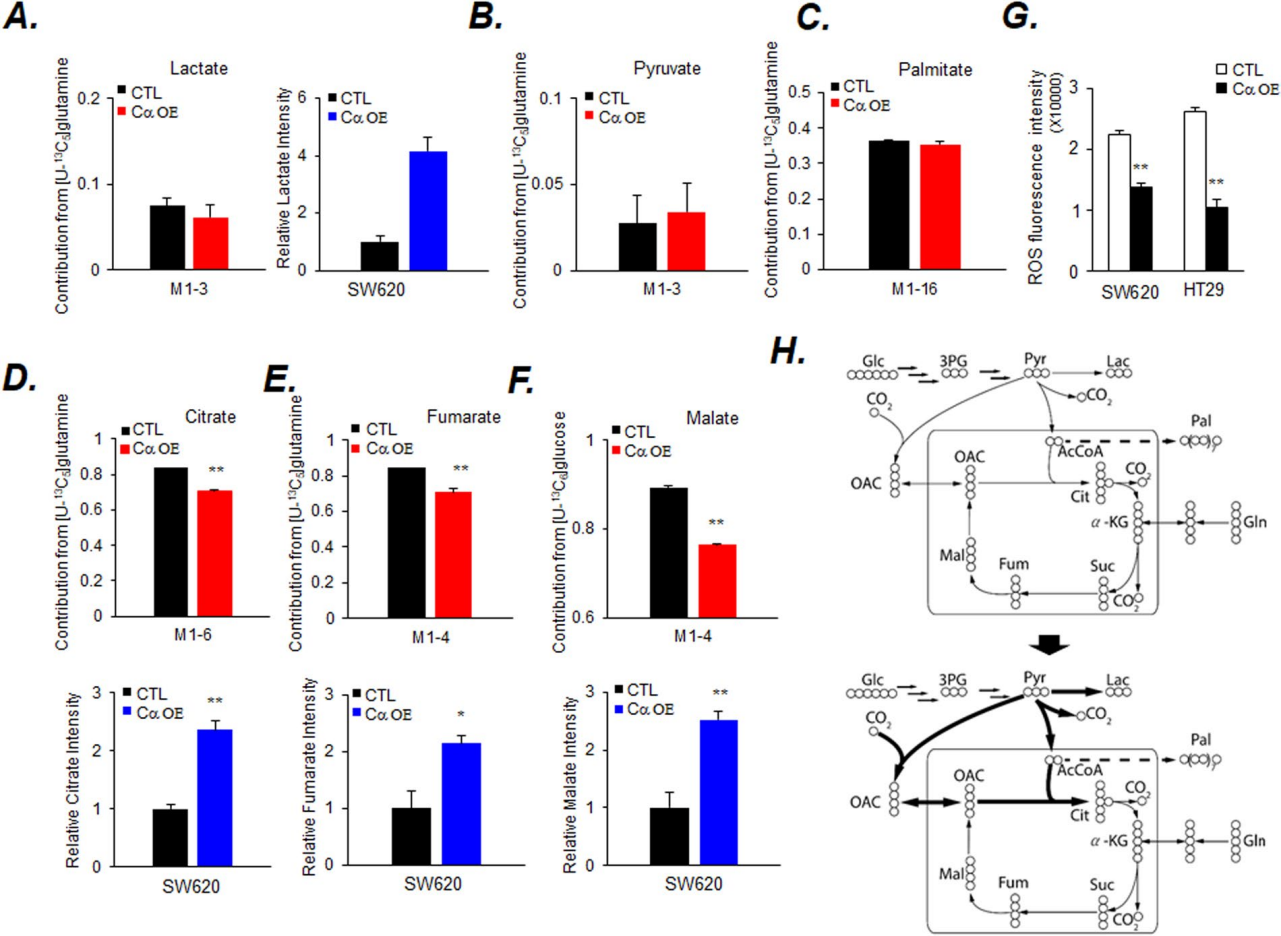
Stable isotope tracer analysis of [U-^13^C]-Gln and metabolic change model. Cells (5 × 10^6^) were cultured in Gln-depleted media containing 4 mM U-^13^C_5_-Gln overnight before extraction. The contribution (red color) and relative intensity (blue color) of the metabolites, (**A**) lactate (M1-3), (**B**) pyruvate (M1-3), (**C**) palmitate (M1-16), (**D**) citrate (M1-6), (**E**) fumarate (M1-4), and (**F**) malate (M1-4) in the cell extracts were measured by GC-EI-MS. Values are expressed as the mean ± SD (n = 5; *p < 0.05; **p < 0.01). (**G**) The levels of ROS were measured by DCFDA staining and analyzed with a microplate reader at an excitation wavelength of 485 nm and emission wavelength of 535 nm. Cells (2 × 10^4^) were cultured for 24 h. Data are presented as a bar graph. Values are expressed as the mean ± SD (n = 3; **p < 0.01). (**H**) Flux map of central carbon metabolism of Cα OE cells predicted by isotope tracer analysis.

We previously observed increased glucose consumption and increased lactate in Cα OE cells^29^. To assess the consumption and release profiles of key cellular metabolites of Cα OE cells more accurately, stable isotope tracer analysis was performed using uniformly labeled [U-^13^C] Glc or Gln. The results of [U-^13^C] Glc analysis in Cα OE cells showed that lactate (M3) and pyruvate (M3) contributions significantly increased (Fig. 2A left B, Supplemental Figs S2A,B, S4A left B and S5A,B), as did the relative lactate production (Fig. 2A right and S4A right). Based on the decrease of the M0 isotopomer of palmitate, the contribution of Glc to lipids seemed to increase (Fig. 2C, Supplemental Figs S2C, S4C and S5C). Contribution to citrate, fumarate, and malate also significantly increased (Fig. 2D–F; top and Supplemental Fig. S2D–F) in SW620-Cα OE cells. The relative citrate intensity increased, the relative intensities of fumarate and malate did show an increase (Fig. 2D–F; bottom). Futhermore, in HT29-Cα OE cells, only the relative intensities of citrate, fumarate, and malate increased (Supplemental Figs S4D–F and S5D–F). The Glc contribution to citrate (M5), particularly, showed a significant increase, indicating the contribution to an oxaloacetate-to-citrate flux (Supplemental Fig. S2D). In addition, the Glc contribution to citrate (M6), showed a significant increase, indicating the contribution to an acetyl CoA flux (Fig. 3H). The contributions to serine (M3) and glycine (M2) increased in both cells (Fig. 2G,H, Supplemental Figs S2G,H, S4G,H and S5G,H). [U-^13^C] Gln analysis revealed no difference between the contributions to lactate (M3) and pyruvate (M3) (Fig. 3A left B, Supplemental Figs S3A,B, S6A left B and S7A,B), whereas the relative lactate intensity increased (Fig. 3A right and S6A right). There was no difference between both cell types in the contribution to palmitate (Fig. 3C and Supplemental Figs S3C, S6C and S7C). The contributions to citrate, fumarate, and malate significantly decreased (Fig. 3D–F; top, Supplemental Fig. S3D–F) and their relative intensities increased (Fig. 3D–F; bottom) in SW620-Cα OE cells. These observations support the proposition that glucose is required in much higher amounts than glutamine to accomplish the total metabolic requirement of SW620-Cα OE cells. Similarly, in HT29-Cα OE cells, only the relative intensities of citrate, fumarate, and malate significantly increased and no difference between the contribution (Supplemental Figs S6D–F and S7D–F). This observation supports the suggestion that glucose and glutamine are required at similar levels to accomplish the total metabolic requirements of HT29- Cα OE cells.

Intracellular ROS level, an indicator of mitochondrial oxidative phosphorylation, markedly decreased in Cα OE cells (Fig. 3G). Utilization of Glc was observed; the results of stable isotope tracer analysis showed that continuous activation of CK2α in colon cancer cell lines accelerated the Warburg effect and lead to the synthesis of an intermediate of the TCA cycle (Fig. 3H and Supplemental Fig. S6G).

### Cα OE-induced LDHA inhibition reduced cell migration and invasion, and tumor growth

**Figure 4:**
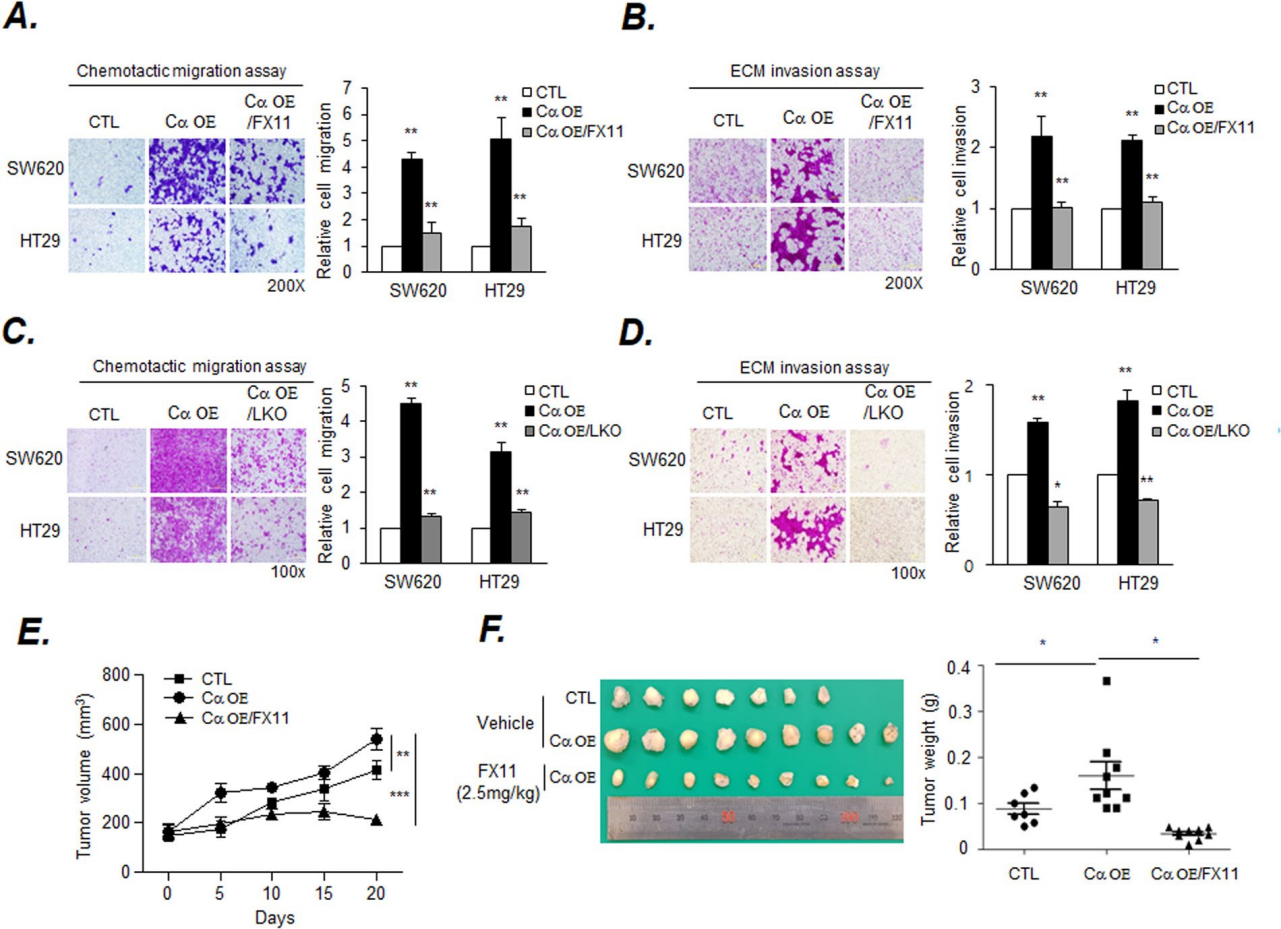
Inhibiting LDHA by FX11 and CRISPR-cas9 decreased Cα OE cells migration and invasion and inhibited xenograft tumor growth. (**A** and **B**) FX11 inhibited the migration and invasion of Cα OE cells. Cells (2 × 10^5^) were exposed to 10 μM FX11 for the indicated time-points. Migration and invasion were assessed by the chemotactic transwell assay. Original magnification, 200×. Values are expressed as the mean ± SD (n = 3; **p < 0.01). Scale bar, 500 µm. (**C** and **D**) Inhibition of migration and invasion by LDHA knockout (KO) in Cα OE cells. *LDHA* KO was performed with the CRISPR-cas9 system. Cells (2 × 10^5^) were incubated for 48 or 72 h. Invasion was assessed by extracellular matrix-coated transwell assay. Original magnification, 100×. Values are expressed as the mean ± SD (n = 3; *p < 0.05; **p < 001). Scale bar, 500 µm. (**E**) Cells (4 × 10^7^ cells/mouse) were injected into the right flank of female nude mice. After tumor establishment (tumor volume of 100–150 mm^3^), the mice were treated with FX11 (2.5 mg/kg body weight) or 2% DMSO as a vehicle. The tumor volume was measured once every five days and expressed as mean volume of tumors (n = 6 or 7 per group; **p < 001; ***p < 0.001). Values are expressed as mean ± S.D. (**F**) Tumors were excised on the 20^th^ day after inoculation and photographed. Quantification of mean tumor weight is presented as a bar graph (n = 6 or 7 per group; *p < 0.05).

Since the Cα OE cells showed an increase in the lactate levels in Fig. 2A, an LDHA inhibitor, FX11, was used to determine whether LDHA expression is required for the CK2-modulated migration and invasion of colon cancer cells. Additionally, LDHA CRISPR/Cas9 was transfected into Cα OE cells for genetic deletion, and the following tests were performed using a well-established clone (Supplemental Fig. S8, blue color). Similarly, under both conditions, migration and invasion were found to be significantly lower in Cα OE cells with LDHA inhibition than in Cα OE cells (Fig. 4A–D). On the other hand, to investigate which mechanism plays a major role in the inhibition of the migration and invasion of Cα OE cells, we treated the cells with the inhibitor or virus for the indicated time-points. The effect of LDH inhibition on the Cα OE cells were investigated using fluorescence-activated cell sorting (FACS) analysis of the DNA content. The results show that the treatments with FX11 and the lentivirus inducing the sub-G1 population and causing minor cell cycle arrest (Supplemental Fig. S9).

To determine whether this finding can be extended to a xenograft model, SW620 (C) and SW620-Cα OE cells were inoculated into nude mice s. c. SW620-Cα OE tumor-bearing mice were intraperitoneally injected with FX11 (2.5 mg/kg body weight) daily for two weeks. The tumor size in mice inoculated with SW620-Cα OE cells were larger than that in mice inoculated with the control cells, and the FX11 treatment significantly reduced tumor growth (Fig. 4E). At the end of the experiments, the tumors were removed from the mice and weighed. Tumor weight of the FX11-treated groups was also reduced, compared to the vehicle-treated SW620-Cα OE inoculated group (Fig. 4F).

### Inhibition of migration and invasion by inducing ROS generation in LDHA knockout (LKO) cells

**Figure 5:**
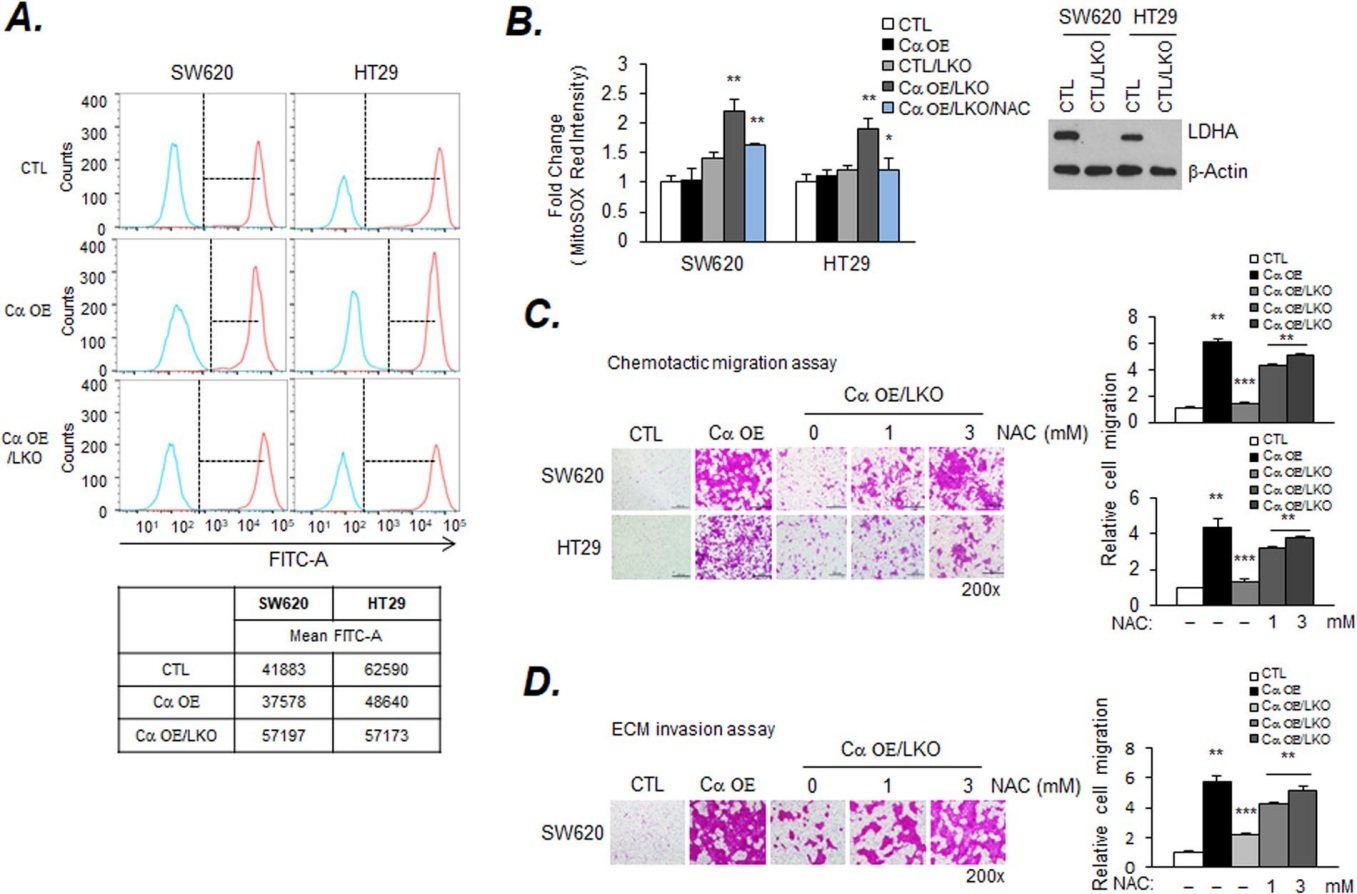
Effect of migration and invasion on lactate dehydrogenase A (LDHA) knockout (KO)-induced ROS production. (**A**) ROS production by LDHA knockout. Cells (5 × 10^5^) were cultured for 24 h. The levels of ROS were measured by DCFDA staining and analyzed by LSRII FACS. (**B**) The levels of ROS were measured by DCFDA staining and analyzed with a microplate reader at excitation wavelength of 485 nm and emission wavelength of 535 nm. Cells (2 × 10^4^) were cultured for 24 h. Data are presented as a bar graph. Values are expressed as the mean ± SD (n = 3; *p < 0.05; **p < 0.01). (**C** and **D**) The reduced migration and invasion were restored by NAC. *LDHA* KO was performed with the CRISPR-cas9 system. Cells (2 × 10^5^) were exposed to 1 and 3 mM NAC for the indicated time-points. Migration and invasion were assessed by the chemotactic transwell assay. Original magnification, 200×. Values are expressed as the mean ± SD (n = 3; **p < 0.01; ***p < 0.001). Scale bar, 500 µm.

It has been reported that LDHA silencing in many cell types increases ROS generation^14^. Whether CK2 is associated with ROS production by LDHA inhibition is unclear. To examine the mechanism by which LKO in Cα OE cells decreases cell migration and invasion, the level of ROS generation was measured. The FACS analysis results showed that the generation of intracellular steady-state ROS decreased in Cα OE cells compared to that in CTL cells, whereas ROS levels increased following LKO (Fig. 5A). To further confirm the contribution of ROS accumulated in mitochondria, mitochondria of living cells were selectively targeted and infiltrated using MtoSOX red, which detects the production of superoxide. In CTL, CTL/LKO, and Cα OE cells, the level of red fluorescence intensity was not significantly different, whereas the intensity of the Cα OE /LKO cell line was significantly increased (Fig. 5B). The fluorescence signal was reduced to a great extent after treatment with the radical scavenger NAC. MitoSOX-fluorescent cells showed consistent results (Supplemental Fig. S10). To confirm the role of ROS in the reduction of migration and invasion by LKO, NAC treatment was administered during the transwell assay. The increased migration and infiltration of Cα OE was markedly reduced after LKO; however, surprisingly, it recovered in cells treated with NAC (Fig. 5C,D). Migration and invasion resulting from CK2 activation were suppressed when only LDHA was blocked. Dimethyl-α-ketoglutarate (DM-α-KG) was utilized to activate energy metabolism, but did not contribute to the migration and invasion recovery of Cα OE/LKO cells (Supplemental Fig. S11).

### Inhibition of LDHA metabolic targets suppresses migration and metastasis

**Figure 6:**
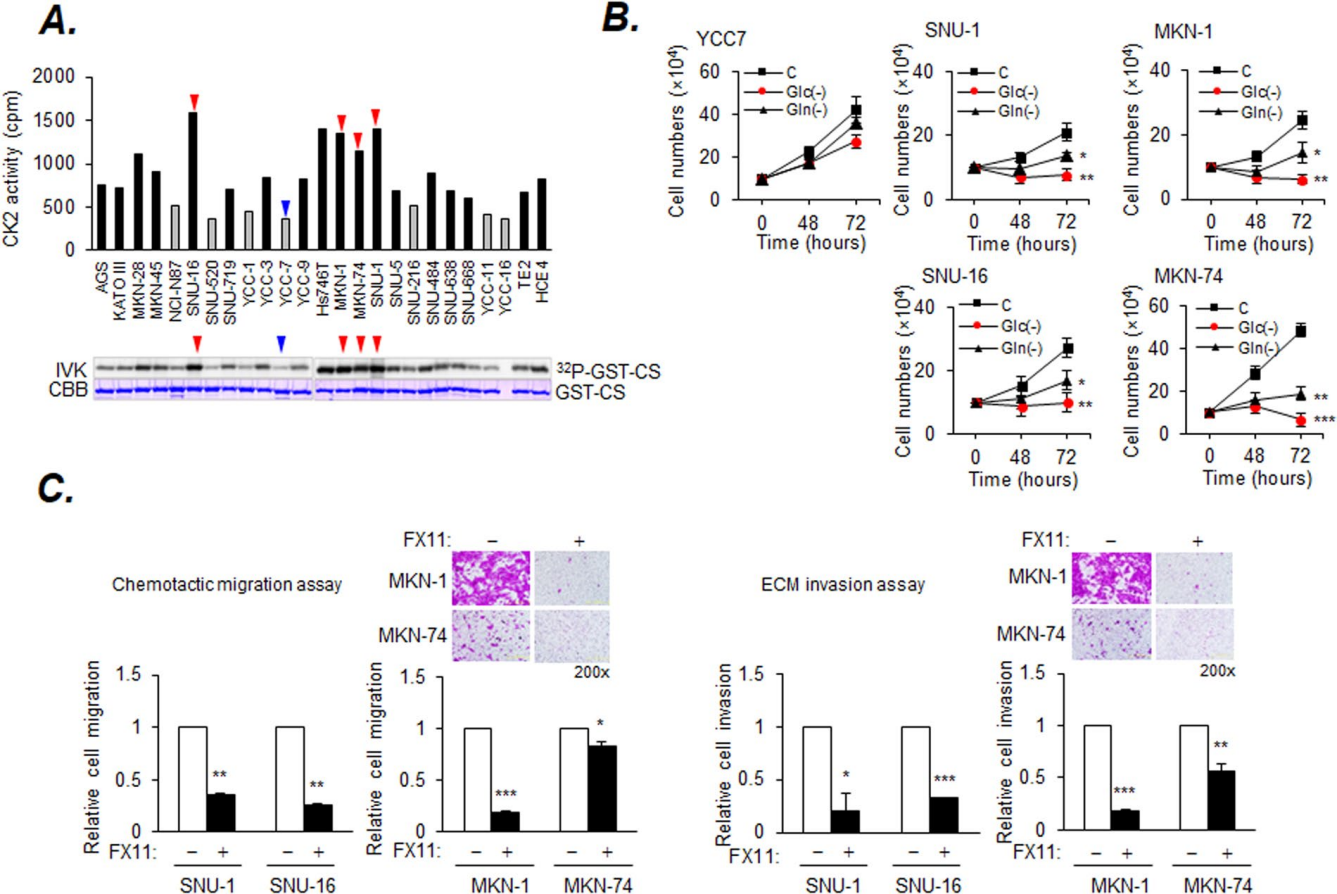
LDHA inhibition reduces cell migration and invasion in cancer cells with high CK2 activity. (**A**) Quantification of CK2 kinase activity in cancer cells. ^32^P-GST-CS (GST-tagged CK2 Substrate) represents ^32^P-labeled GST-CS and CBB represents Coomassie blue-stained input GST-CS, respectively. (**B**) The number of cells was counted using an ADAM automatic Cell Counter. Cells (1 × 10^5^) were incubated in Glc- or Gln-free RPMI and the number of surviving cells was estimated at the indicated time-points. (**C**) Reduced migration and invasion by FX11. Cancer cells (2 × 10^5^) were exposed to 10 μM FX11 for 72 h. Migration and invasion were assessed by the chemotactic transwell assay. Original magnification, 200×. Values are expressed as the mean ± SD (n = 3; *p < 0.0; **p < 0.01; ***p < 0.001). Scale bar, 500 µm.

To assess the effect of LDHA expression according to intrinsically high CK2 activity on cell migration and invasion, CK2 kinase activity was measured in various gastric cancer cell lines (Fig. 6A). The MKN-1, MKN-74, SNU-16, and SNU-1 cell lines were chosen because CK2 activity and LDHA expression were high in these cell lines (Supplemental Fig. S12). To assess the nutritional requirements of these cells with regards to a carbon source, cell growth was monitored under Glc- and Gln-depletion conditions. The numbers of SNU-1, SNU-16, MKN-1, and MKN74 cancer cells showing high levels of CK2 activity were notably reduced after 72 h of culture under Glc-depleted conditions as compared to the ones cultured under Gln-depleted conditions. The number of YCC7, SNU-1, SNU-16, and MKN-1 cells were moderately reduced and the number of MKN-74 cells was significantly reduced (Fig. 6B). The numbers of migrated and invaded MKN-1 and MKN-74 cells were reduced by FX11 (Fig. 6C). In addition, migration and invasion were also markedly reduced by LKO; they increased again when the cells were treated with NAC, a ROS scavenger (Supplemental Fig. S13).

## Discussion

CK2 regulates the glucose metabolic pathway of bladder cancer cells^24^, enhances tumor cell motility in head and neck cancer cells^30^, and facilitates the invasion ability of colon cancer cells^31^. Notably, elevated CK2 activity is associated with malignant transformation^32^. We observed excessive glucose consumption and lactate production in Cα OE cells. However, the network and mechanism by which CK2α regulates the migration and invasion of cancer cells after they are subjected to metabolic modifications is unclear.

In the present study, using isotope tracer analysis, we demonstrated that Cα OE cells facilitated glucose utilization for supporting cell proliferation (Fig. 1). Proliferating Cα OE cells increased the contribution to pyruvate (M3) and citrate (M3~6) via oxaloacetate (Fig. 3H and Supplemental Fig. S6G). These cells had decreased growth and colony formation abilities under Glc-deprivation conditions. We also found that increased LDHA in the modified metabolic pathway axis, driven by CK2α, regulates cancer cell migration and invasion (Fig. 1).

In glycolytic cancer cells, Glc acted as a fuel for survival^6^, and Glc depletion induced apoptosis^33^. Compared to the case under Gln-depletion conditions, under Glc-depletion conditions, when oncogenic CK2α was overexpressed, the reduction in the number of surviving cells was larger than that of CTL cells. Additionally, the colony-forming ability, migration, invasion, and number of surviving cells decreased considerably (Fig. 1). Additionally, the number of dead cells increased in this condition (Fig. 1B). A recent report showed that Glc and Gln support oncogenic transformation by maintaining invasive cancer phenotypes^6,7^. However, according to our results, Glc was found to be more important than Gln as a carbon source, for survival, migration, and invasion, particularly in cells expressing high levels of CK2α.

A metabolic change can be used to evaluate the multiplication, survival, and eventually, metastasis of cancer cells. We traced uniformly labeled carbon sources to understand the manner in which aerobic decomposition and other metabolic changes observed in cancer cells support the diverse requirements of cell migration and metastasis more accurately. In comparison to CTL cells, the ^13^C_6_-Glc contribution was higher than the ^13^C_5_-Gln contribution in Cα OE cells. SW620-Cα OE cells may facilitate both the Warburg effect and minimally use glutamine in TCA cycle flux, depending on the cell type (Fig. 3H and Supplemental Fig. S6G). In the HT29-Cα OE cells, the ^13^C_6_-Glc contribution showed an increase in the glycolysis pathway (Supplemental Figs S4 and S5) but that of the TCA intermediates was unchanged (Supplemental Fig. S4D–F); there was no difference in the ^13^C_5_-Gln contribution, but the relative intensities of lactate, citrate, fumarate, and malate increased significantly (Supplemental Figs S6 and S7). Feeding Glc into the TCA cycle via acetyl CoA is very important for cancer cell proliferation. Alternatively, the conversion of oxaloacetate from pyruvate into the TCA cycle further enhances cancer cell growth rapidly. Although both cells have different degrees of glutamine utilization, Cα OE cells commonly promoted this pathway. Pyruvate carboxylase (PC) is known to be involved in this pathway. Silencing of PC expression and activity impairs NSCLC cell growth, tumor formation and antioxidant capacity. Although this phenotypic effect of PC inhibition is related to the important metabolic function of PCs in non-small-cell lung cancer, including mitochondrial cycle activity, and nucleotide and redox homeostasis^34^, further investigations regarding the functional properties and metabolic features of PC are needed.

In a previous report, we found that LDHA expression increased through the CK2/PKM2/LDHA axis^29^. In this study, the results from isotope tracer analysis supported that high levels of CK2 drive the Warburg effect in the cells, causing an increase in the lactate generation by LDHA. Similarly, recent reports have suggested that high levels of lactate derived from aerobic glycolysis not only promote the destruction of adherence junctions, but also increase the metastatic potential by the induction of extracellular acidification^35^. An acidic pH was also reported to induce tumor cell motility and suppress cytokine release and monocyte migration; thus, it is linked to metastasis, tumor recurrence, and poor prognosis in some cancer patients^36,37^. Our results also changed to acid pH in the Cα OE cells more rapidly than control cells (data not shown). In our present results, the abilities of migration and invasion of Cα OE cells increased, which were suppressed by FX11, an LDHA inhibitor (Fig. 4).

Mitochondrial oxidative phosphorylation is a major source of ROS generation^38^. The inhibition of LDHA was reported to induce oxidative stress and suppress tumor progression^14^. Our results show that the amplification of the conversion from pyruvate to oxaloacetate by overexpression of CK2 leads to citrate accumulation and promotes LDHA level. As previous reports suggested, shRNA-mediated knockdown of LDHA, a key mediator of aerobic glycolysis, promotes the production of ROS in mitochondria and reduces proliferation and motility^13^. Our results also showed that ROS generation increased after LKO, followed by the reduction of migration and invasion, which were increased again by NAC (Fig. 5). DM-α-KG was supplied as an energy donor of the TCA cycle in this cell, but migration and invasion failed probably because ROS had a functional effect on the cellular pathway (Supplemental Fig. S11). Various cancer cells with high CK2 kinase activity showed Glc requirement and increased endogenous LDHA expression (Fig. 6). Reduction of migration and invasion via LDHA inhibition by FX11 is considered a potentially attainable and acceptable therapy for CK2α-dependent tumors.

In conclusion, we proposed that high levels of CK2α reprogrammed cells to preferentially utilize glycolytic metabolism; this was confirmed by carbon labeling experiment using ^13^C-Glc or ^13^C-Gln as stable isotope tracers. The high expression of LDHA following these metabolic alterations increased the migration and invasion of cancer cells. Our present results provide a critical example that abnormalities occurring at the genetic level in a specific protein such as CK2α, make cancer cells alter their cellular carbon metabolism, which is considered a therapeutic target pathway. Finally, these results demonstrate for the first time that CK2α mediates LDHA expression and increases migration as well as invasion in human cancer cells. The CK2-LDHA axis, therefore, shows promise as an excellent metabolic target for cancer therapy.

## Supplementary information

Supplementary information
