# Supplementary material for: Protein kinase CK2-dependent aerobic glycolysis-induced lactate dehydrogenase A enhances the migration and invasion of cancer cells
Source: Sci Rep. 2019 Mar 29;9:5337. doi: 10.1038/s41598-019-41852-4 (PMC6441004; doi:10.1038/s41598-019-41852-4)

Protein kinase CK2-dependent aerobic glycolysis-induced lactate dehydrogenase A enhances the migration and invasion of cancer cells

Dae-Kyun Im [1], Heesun Cheong [2], Jong Suk Lee[3], Min-Kyu Oh [1,*], Kyung Mi Yang [4,*]

Figure Legend for Supplemental Figures.

Supplemental Figure S1. Glucose depletion efficiently induces Cα OE cell death. (A) SW620 or (B) HT29 cells were cultured in Glc- or Gln-free DMEM and cell death was assessed by Annexin-PI staining at the 72-h time-point. Representative flow cytometry plots are shown. Q1: Viable cells; Q2: Apoptotic cells; Q3: Dead cells; Q4: Necrotic cells

Supplemental Figure S2. Glucose contributes to glycolysis in SW620-Cα OE cells. The contribution of the metabolites, (A) lactate, (B) pyruvate, (C) palmitate, (D) citrate, (E) fumarate, (F) malate, (G) serine, and (H) glycine in the cell extracts was measured by GC-EI-MS.

Supplemental Figure S3. Glutamine contributes to glycolysis in SW620-Cα OE cells The contribution of the metabolites, (A) lactate, (B) pyruvate, (C) palmitate, (D) citrate, (E) fumarate, and (F) malate in the cell extracts was measured by GC-EI-MS.

Supplemental Figure S4. Stable isotope tracer analysis of [U-^{13}C]-Glc in HT29-Cα OE cells. Cells (5 × 10^6) were cultured in Glc-depleted media containing 1 g/L U-$^{13}C_6$-Glc overnight before extraction. The contribution (red color) and relative intensity (blue color) of the metabolites, (A) lactate (M3), (B) pyruvate (M3), (C) palmitate (M1-16), (D) citrate (M3-

6), (E) fumarate (M2-4), (F) malate (M1-4), (G) serine (M1-3), and (H) glycine (M1-2) in the cell extracts were measured by GC-EI-MS. Values are expressed as the mean ± SD (n=5;$*p < 0.05$; $**p < 0.01$).

Supplemental Figure S5. Glucose contributes to glycolysis in HT29-Cα OE cells. The contribution of the metabolites, (A) lactate, (B) pyruvate, (C) palmitate, (D) citrate, (E) fumarate, (F) malate, (G) serine, and (H) glycine in the cell extracts was measured by GC-EI-MS.

Supplemental Figure S6. Stable isotope tracer analysis of [U-^{13}C]-Glc and metabolic change model in HT29-Cα OE cells. Cells (5×10^6) were cultured in Gln-depleted media containing 4 mM U-$^{13}C_5$-Gln overnight before extraction. The contribution (red color) and relative intensity (blue color) of the metabolites, (A) lactate (M1-3), (B) pyruvate (M1-3), (C) palmitate (M1-16), (D) citrate (M1-6), (E) fumarate (M1-4), and (F) malate (M1-4) in the cell extracts were measured by GC-EI-MS. Data are presented as bar graphs. Values are expressed as the mean ± SD (n=5;$**p < 0.01$; $***p < 0.001$). (G) Flux map of central carbon metabolism Cα OE cells predicted by isotope tracer analysis.

Supplemental Figure S7. Glutamine contributes to glycolysis and TCA cycle in HT29-Cα OE. The contribution of the metabolites, (A) lactate, (B) pyruvate, (C) palmitate, (D) citrate, (E) fumarate, and (F) malate in the cell extracts was measured by GC-EI-MS.

Supplemental Figure S8. Knockout of *LDHA* in Cα OE cells. Western blot analysis was performed using the indicated antibodies.

Supplemental Figure S9. Cell cycle distribution of Cα OE cells treated with FX11 and LDHA knockout. Cells (3×10^5) were exposed to 10 μM FX11 or transiently infected with the lentiviral system of *LDHA* KO for 48 h. (A, B) Cell cycle assessed by LSRII FACS analysis. Histogram representation of cell cycle distribution. (C, D) Bar graph representation of cell cycle populations: Sub-G1 (red columns); G1 (block columns); S (Grey column); G2/M (hatched).

Supplemental Figure S10. LDHA knockout causes mitochondrial dysfunction. (A) Visualization of ROS by superoxide-specific indicator MitoSOX red. Treatment with 5 mM NAC was associated with ROS production (n = 3). Scale bar, 500 μm.

Supplemental Figure S11. Reduced migration and invasion by ROS was not regulated by DM-α-KG. (A, B) Cells (2×10^5) were exposed to 3 mM NAC or 10 mM Dimethyl-α-ketoglutarate (DM-α-KG) for 12 h. Migration and invasion were assessed by the chemotactic transwell assay. Original magnification, 200×. Values are expressed as the mean ± SD (n=3; *$p < 0.05$; **$p < 0.01$; ***$p < 0.001$). Original magnification, 200×. Scale bar, 500 μm.

Supplemental Figure S12. LDHA expression is increased in gastric cancer cells with high CK2 activity. LDHA expression levels were assessed by Western blot analysis.

Supplemental Figure S13. Inhibition of migration and invasion by LDHA knockout was restored by NAC. (A) LDHA expression levels were assessed by Western blot analysis. (B, C) The reduced migration and invasion were restored by NAC. *LDHA* KO was performed with the CRISPR-cas9 system. Cells (2×10^5) were exposed to 3 mM NAC for the indicated time-

points. Migration and invasion were assessed by the chemotactic transwell assay. Original magnification, 200×. Values are expressed as the mean ± SD (n=3; *p < 0.01; **p < 0.01). Scale bar, 500 μm.

Supplementary Figure S1

A.

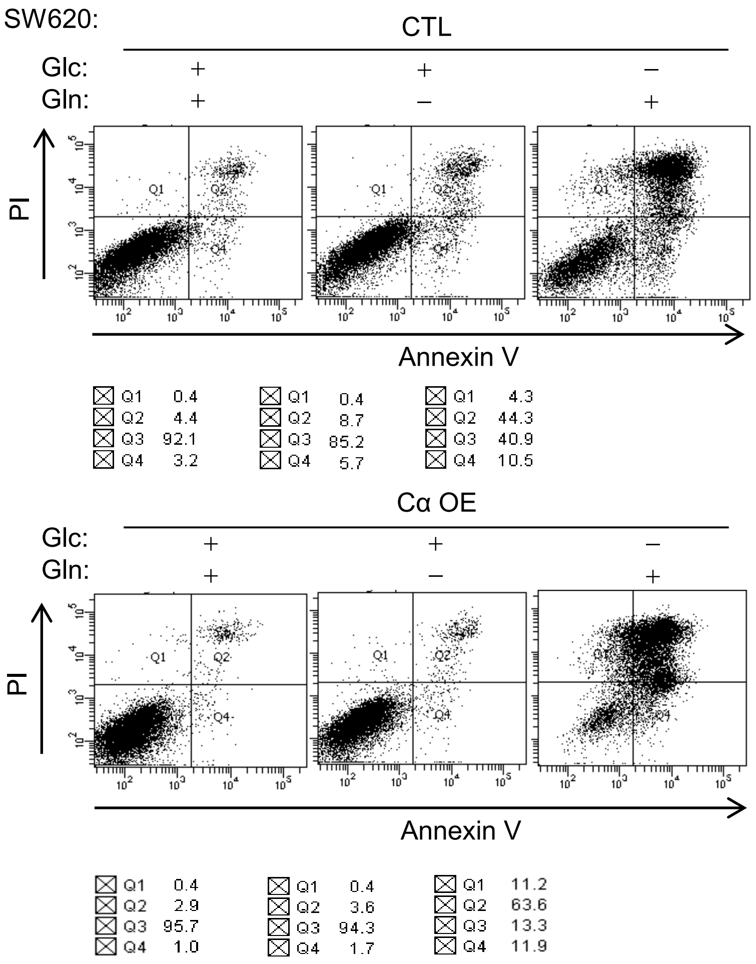

B.

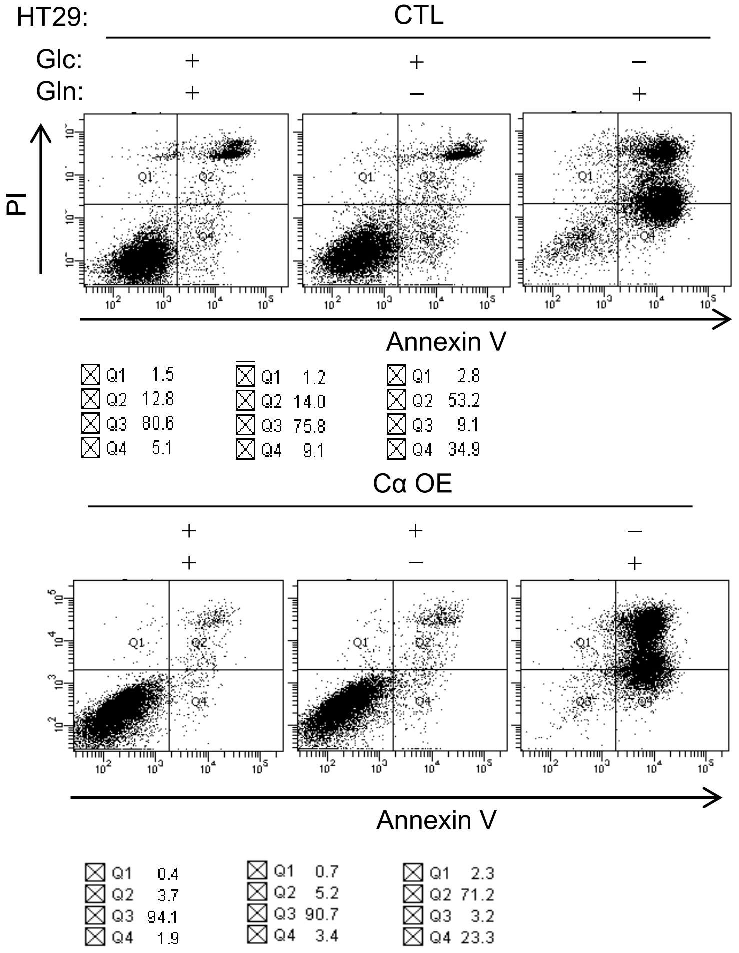

Supplementary Figure S2

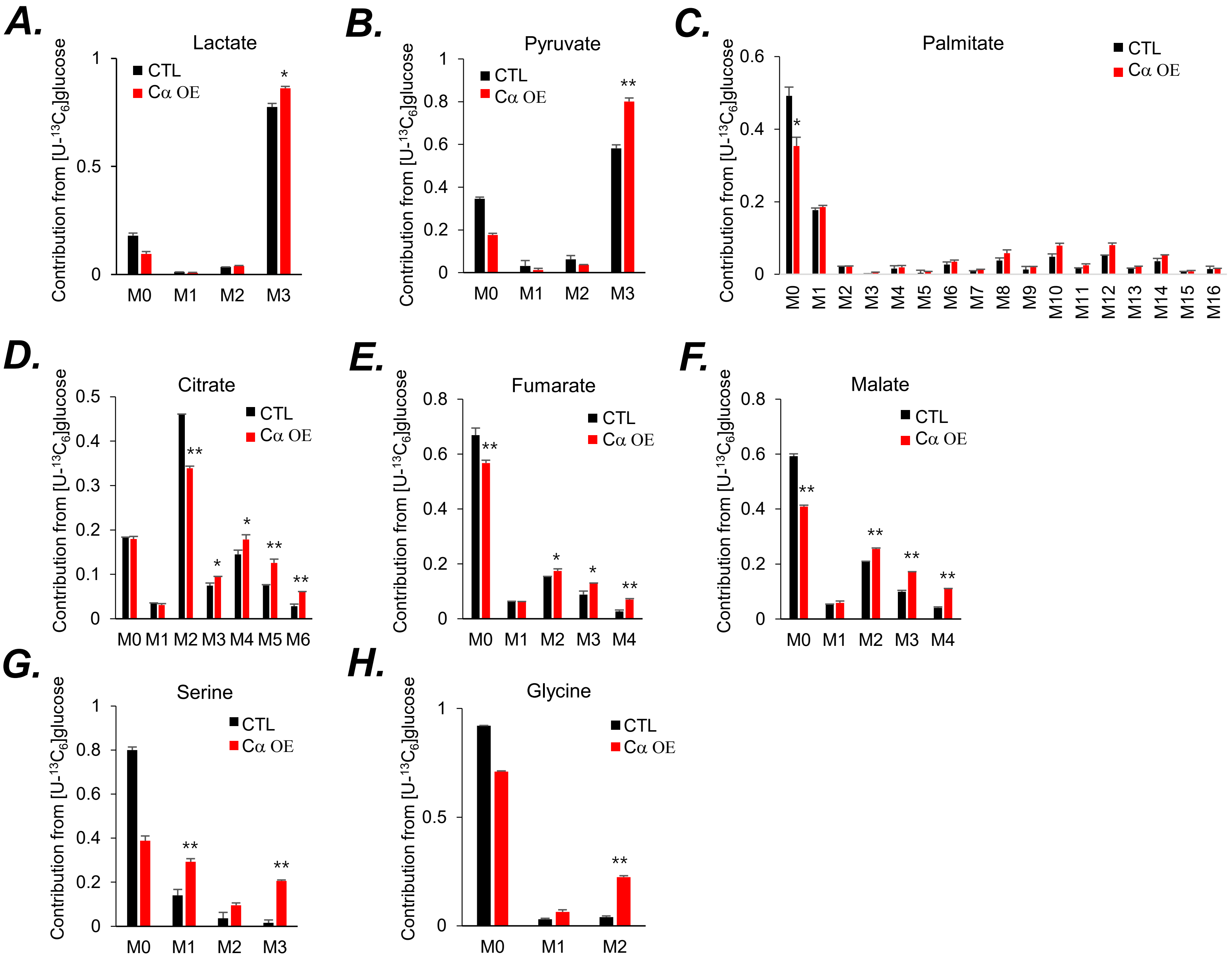

Supplementary Figure S3

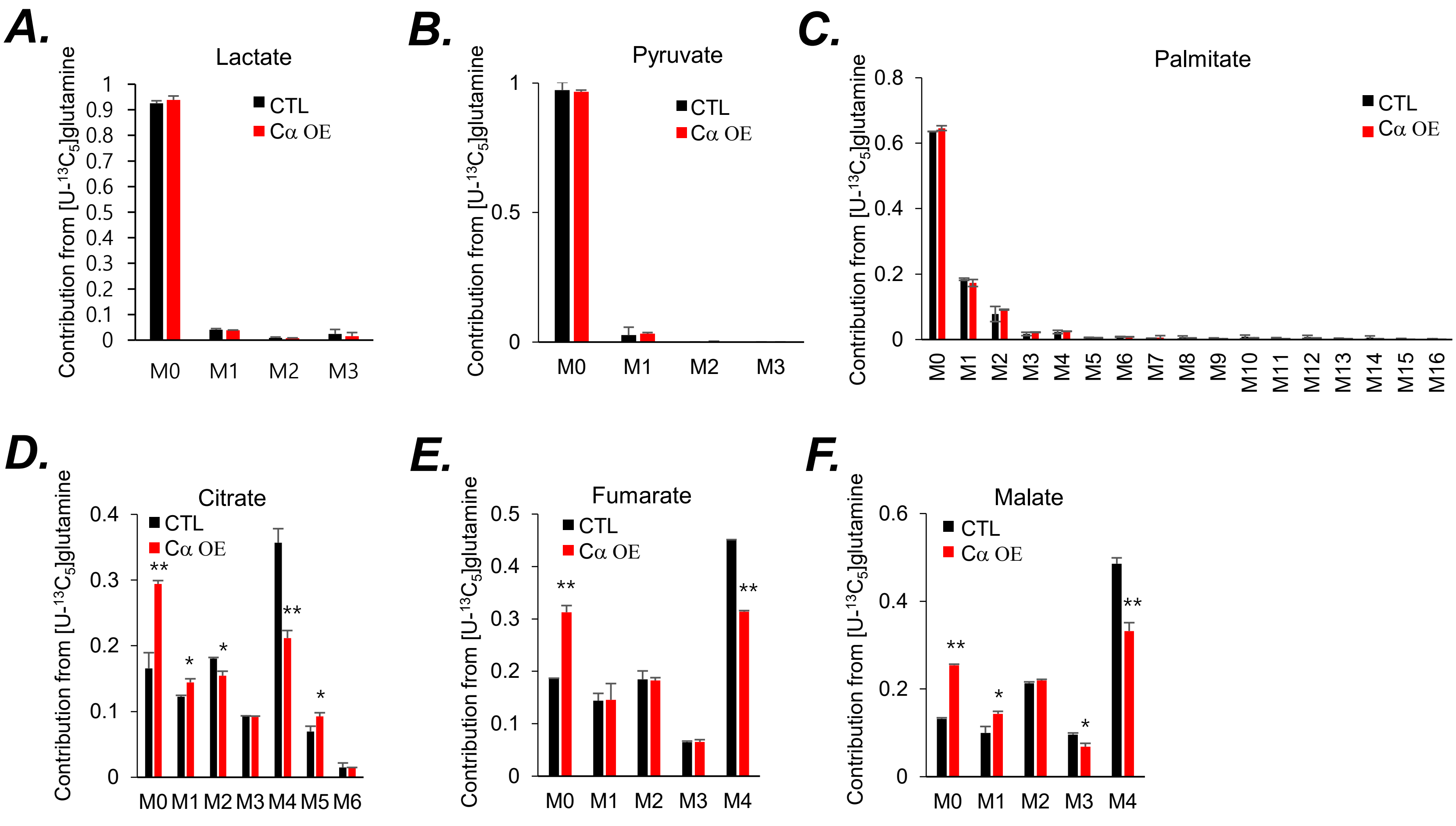

Supplementary Figure S4

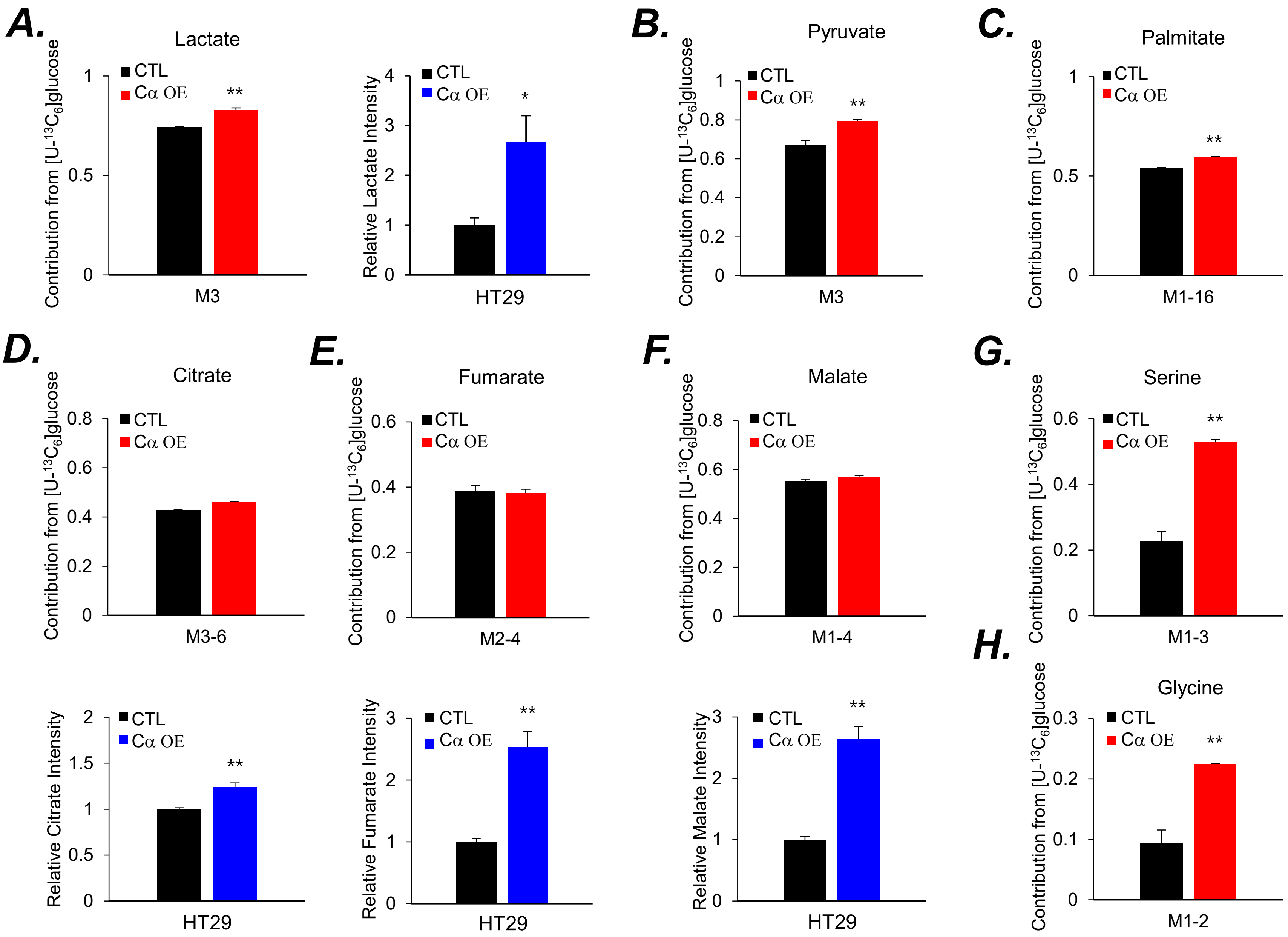

Supplementary Figure S5

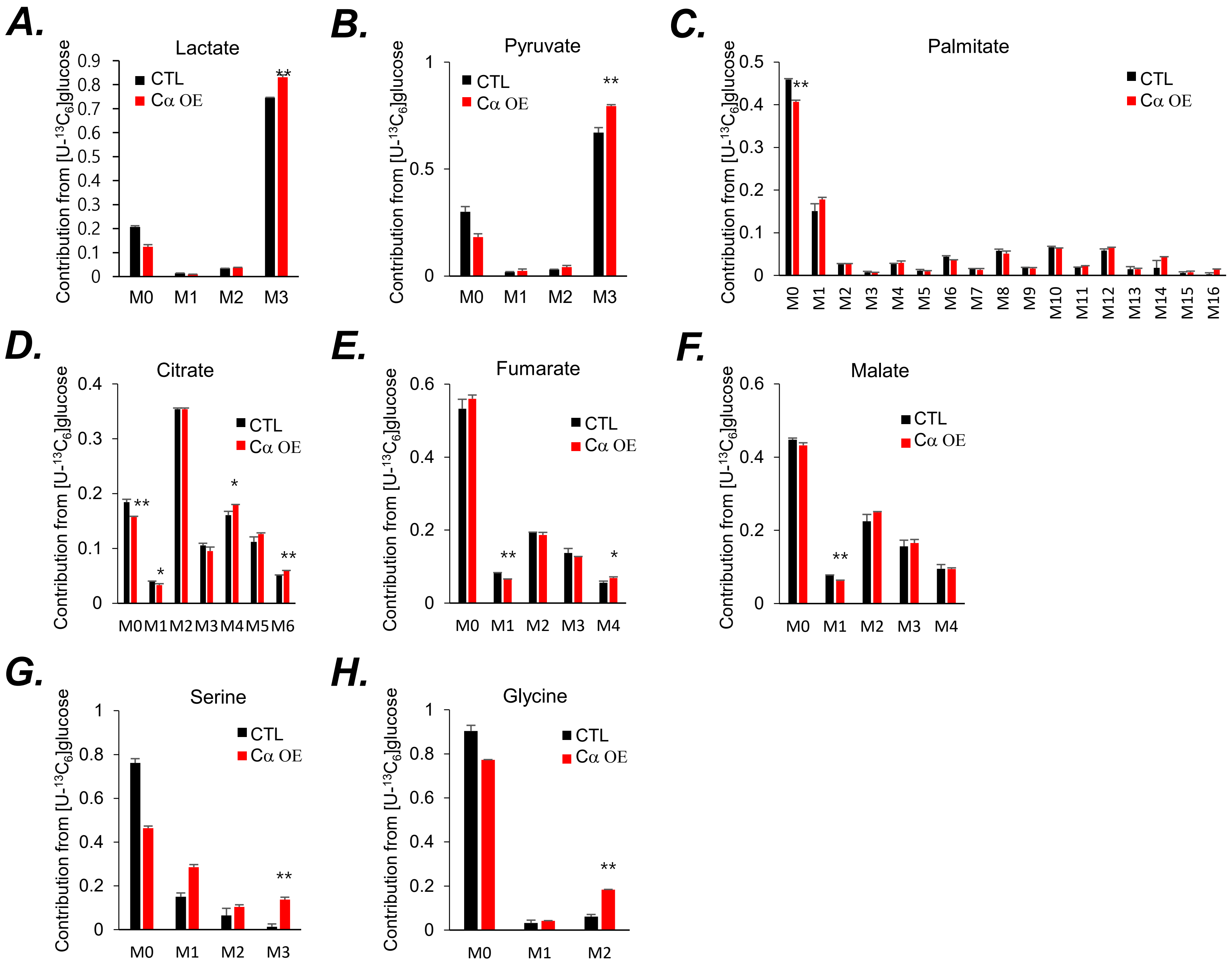

Supplementary Figure S6

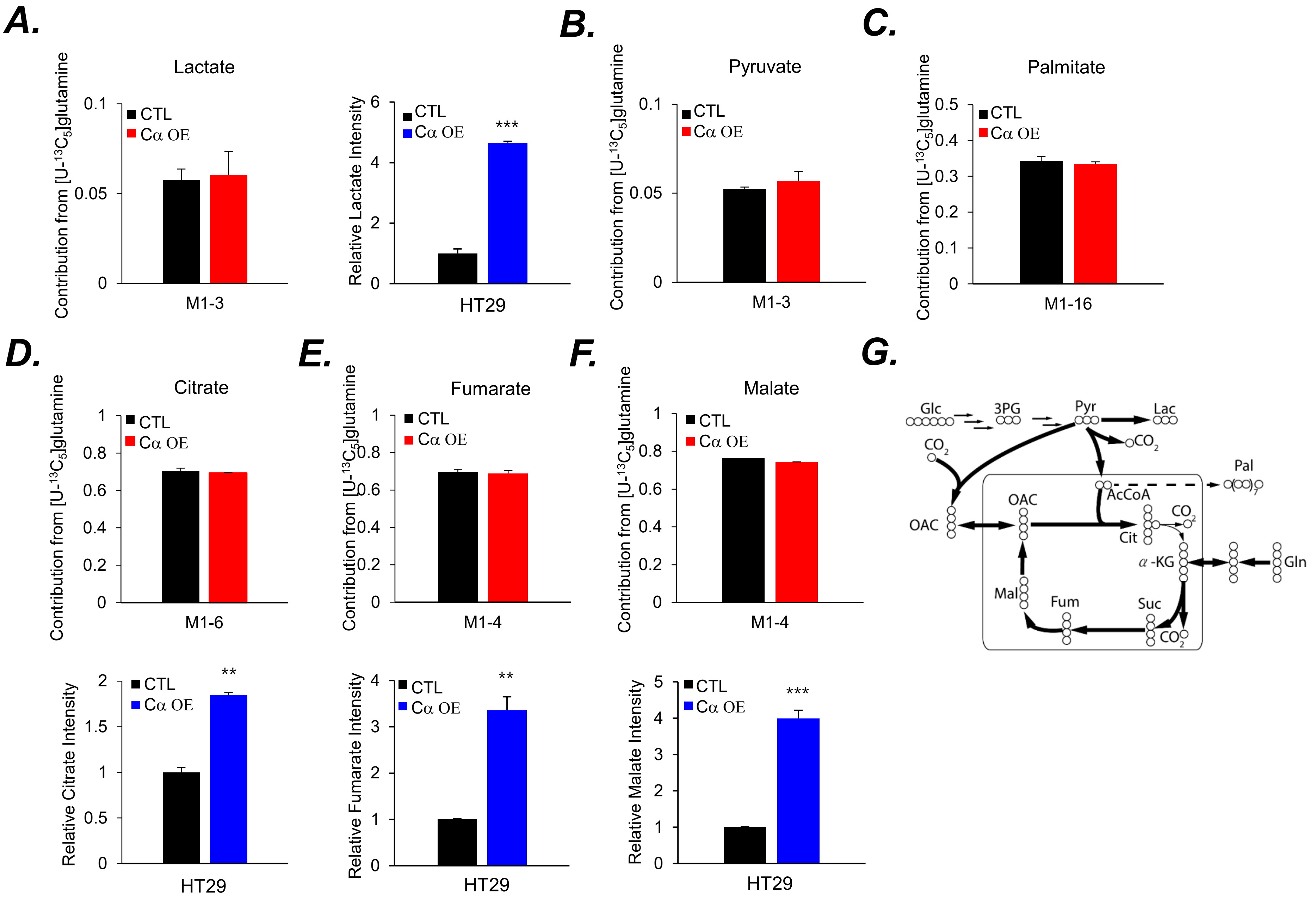

Supplementary Figure S7

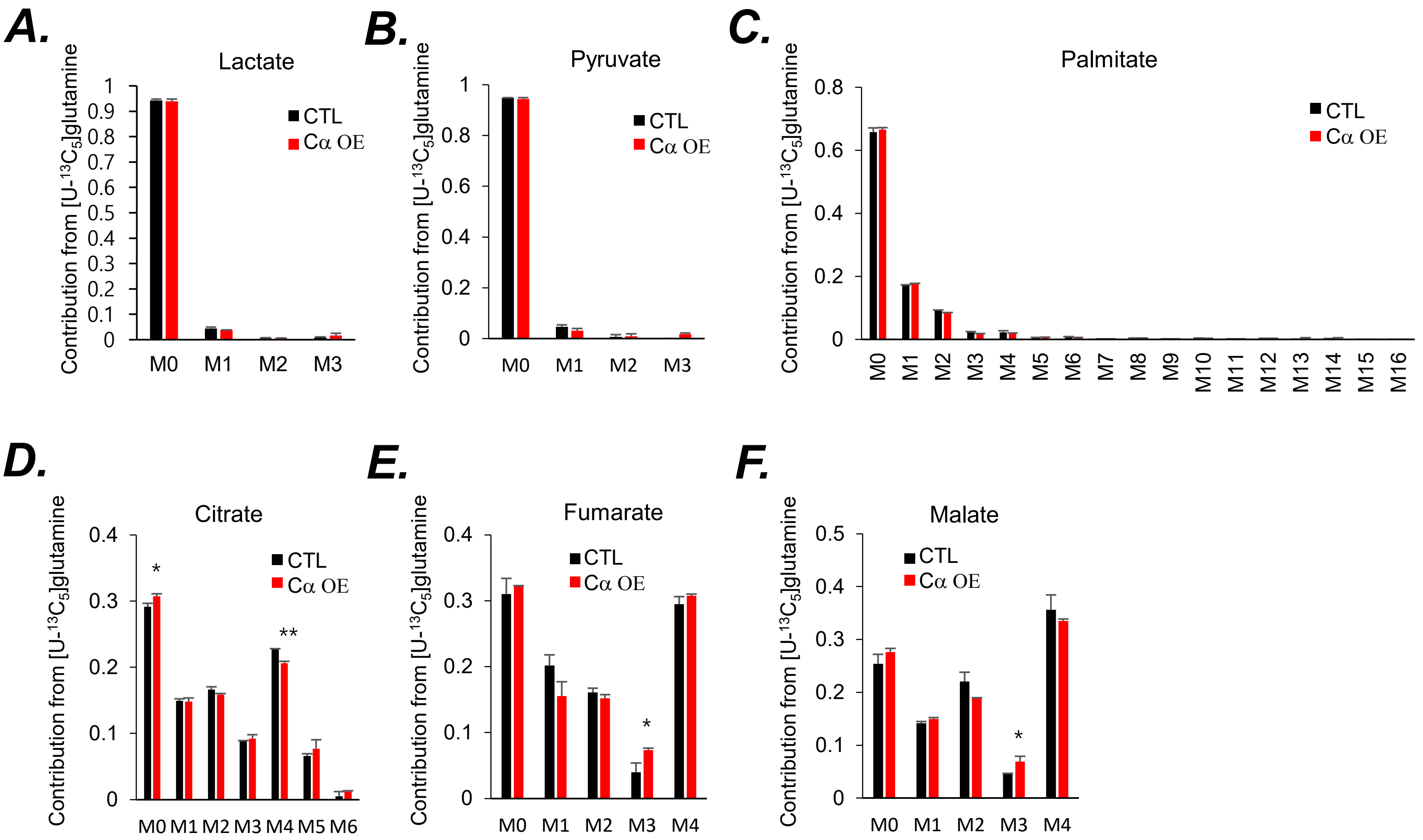

Supplementary Figure S8

A.

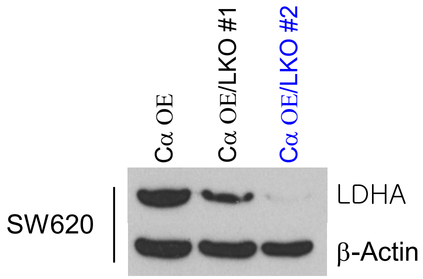

B.

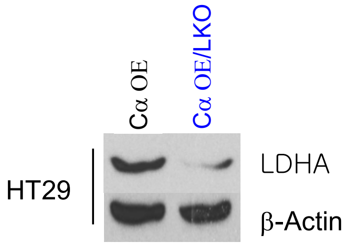

Supplementary Figure S9

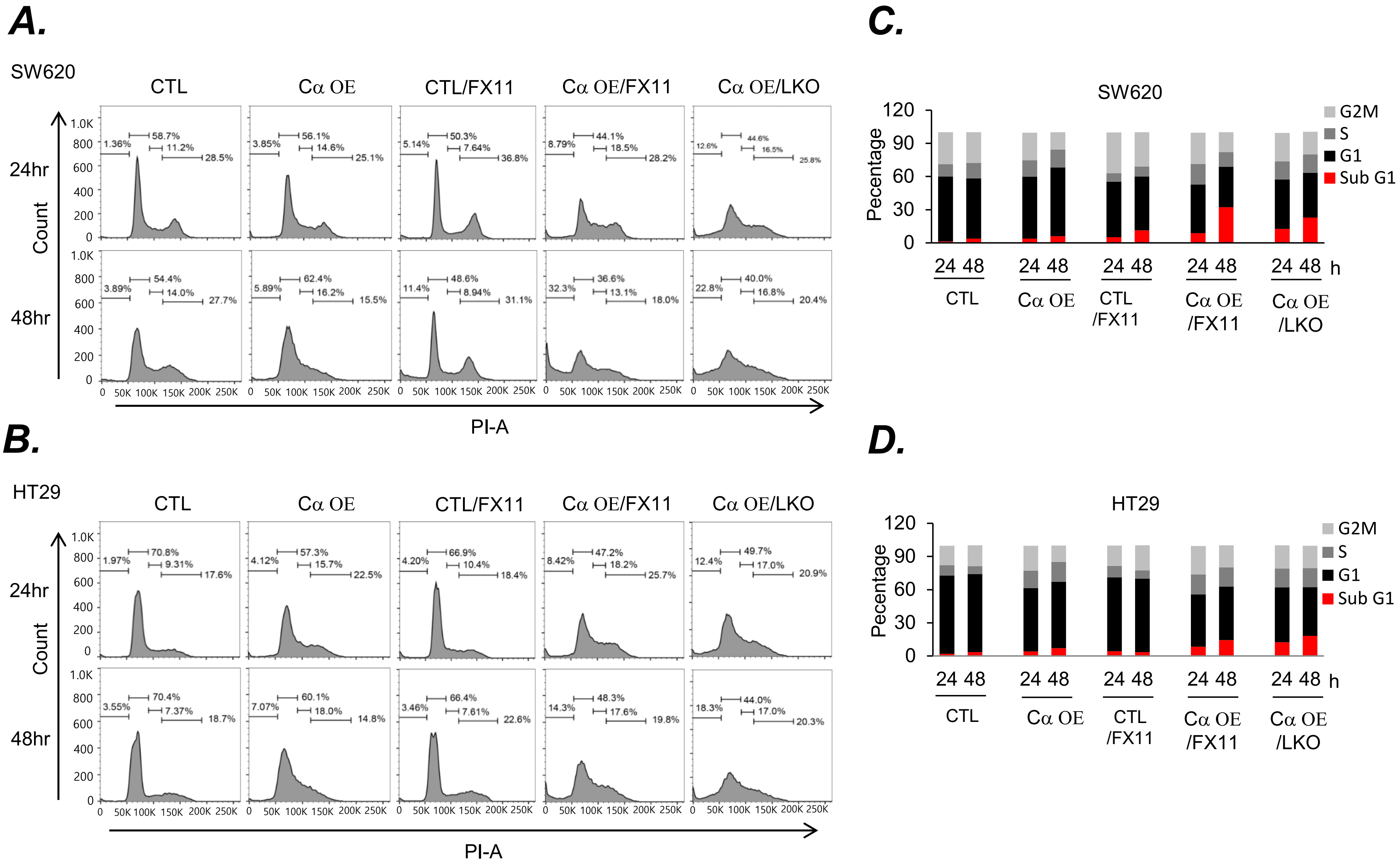

Supplementary Figure S10

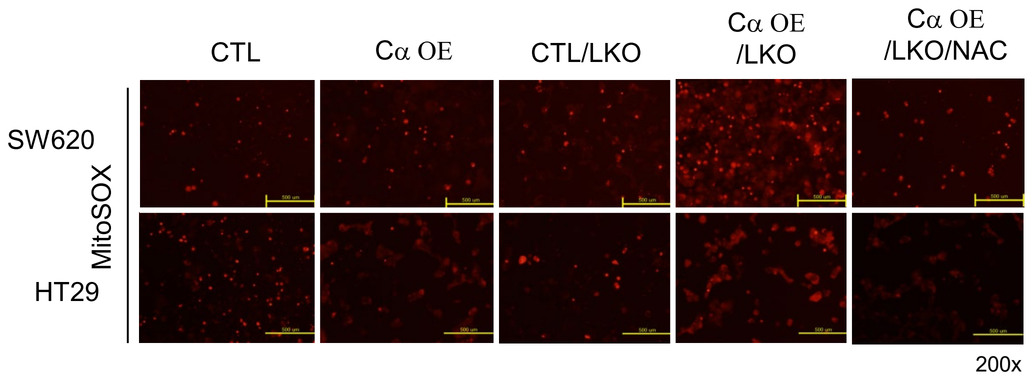

Supplementary Figure S11

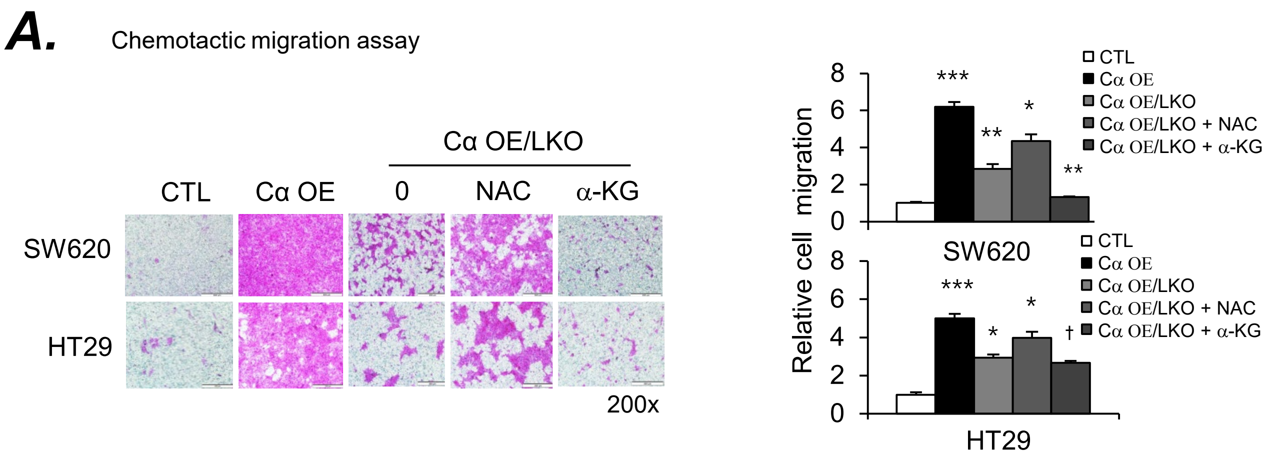

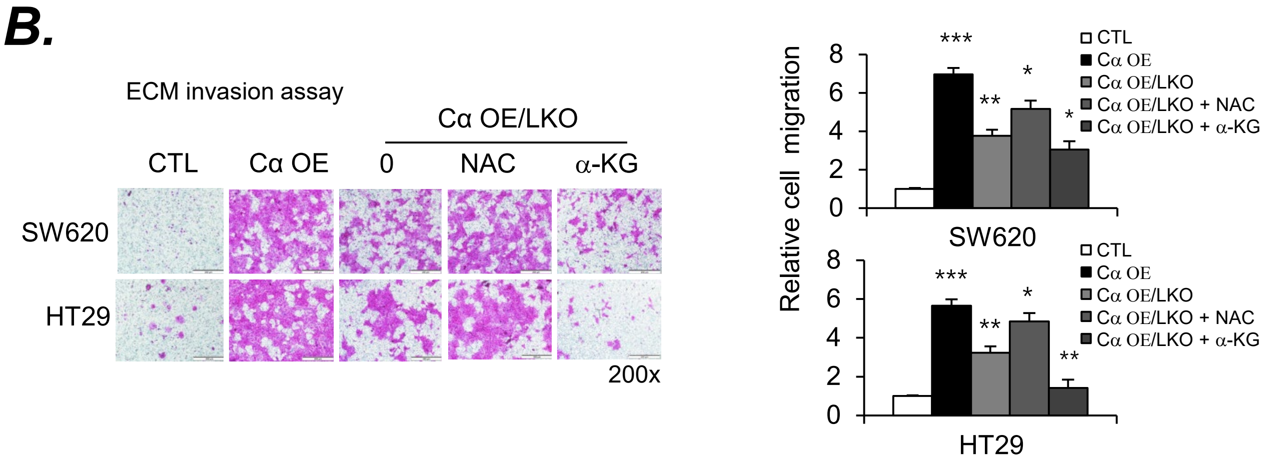

Supplementary Figure S12

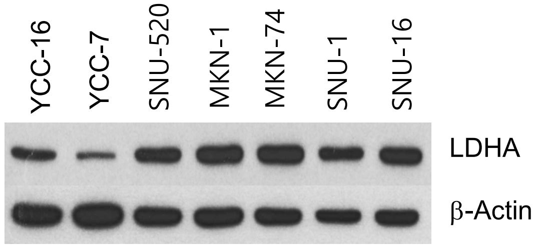

Supplementary Figure S13

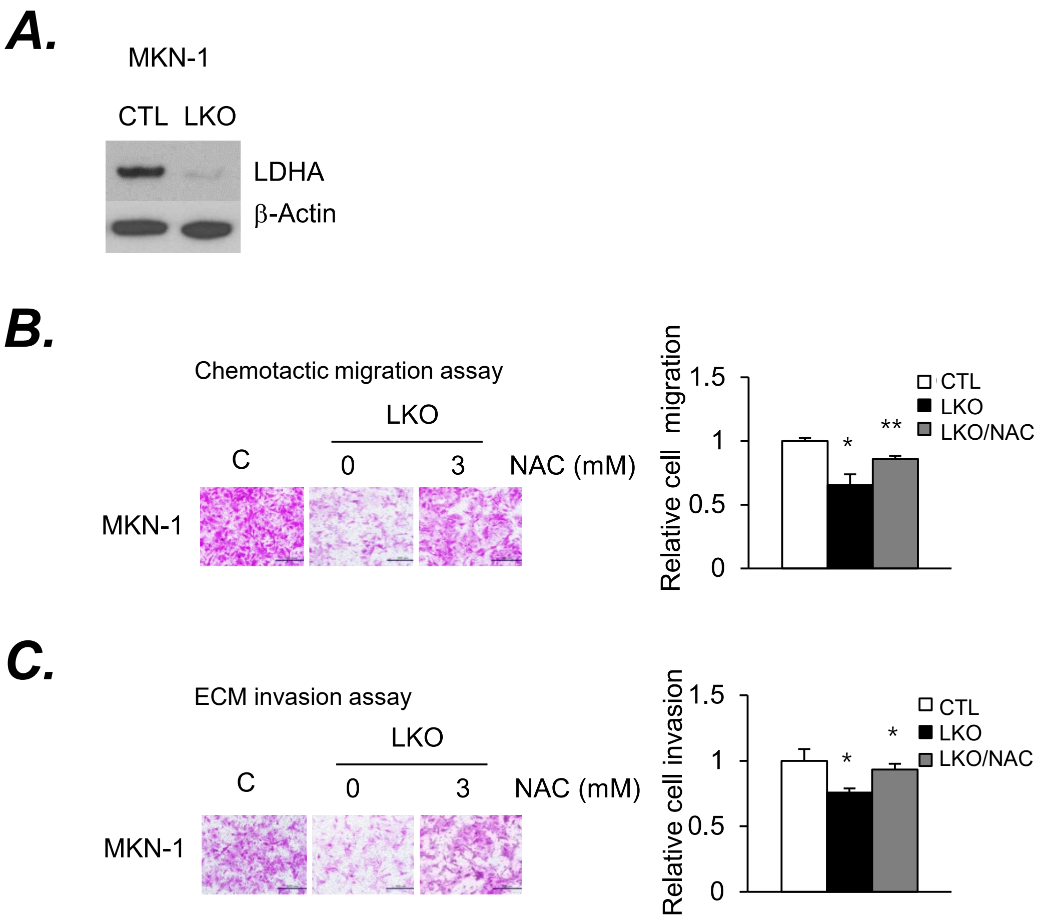

Supplement: Supplementary file 1 — Supplementary information [file 41598_2019_41852_MOESM1_ESM.pdf]
